# Reduced susceptibility to triclosan and proposed epidemiological cut-off values for disinfectants and heavy metals in Escherichia coli isolated from Thai tilapia farms: Implications for One Health antimicrobial resistance surveillance

**DOI:** 10.14202/vetworld.2026.3836-3849

**Published:** 2026-08-30

**Authors:** Varangkana Thaotumpitak, Jarukorn Sripradite, Saran Anuntawirun, Nawaphorn Roongrojmongkhon, Woranich Hinthong, Saharuetai Jeamsripong

**Affiliations:** 1 Department of Microbiology, Faculty of Public Health, Mahidol University, Bangkok 10400, Thailand.; 2 Department of Social and Applied Science, College of Industrial Technology, King Mongkut's University of Technology North Bangkok, Bangkok 10800, Thailand.; 3 Department of Veterinary Public Health, Faculty of Veterinary Science, Chulalongkorn University, Bangkok 10330, Thailand.; 4 Princess Srisavangavadhana Faculty of Medicine, Chulabhorn Royal Academy, Bangkok 10210, Thailand.; 5 Research Center on Clinical and System Microbiology (RCSyM), Chulabhorn Royal Academy, Bangkok 10210, Thailand.; 6 Research Unit in Microbial Food Safety and Antimicrobial Resistance, Department of Veterinary Public Health, Faculty of Veterinary Science, Chulalongkorn University, Bangkok 10330, Thailand.

**Keywords:** antimicrobial resistance, biofilm formation, disinfectant resistance, Escherichia coli, heavy metals, One Health, tilapia aquaculture, triclosan

## Abstract

**Background and Aim:**

Tilapia aquaculture systems may act as reservoirs of antimicrobial resistance (AMR) because continuous exposure to antibiotics, disinfectants, and heavy metals can select for resistant bacterial populations. Although *Escherichia coli* is widely recognized as an indicator organism for monitoring environmental AMR, information on reduced susceptibility to disinfectants and heavy metals in Southeast Asian aquaculture remains limited. Furthermore, epidemiological cut-off values (ECOFFs) for these agents have not been established for *E. coli* isolated from Thai tilapia farms. This study aimed to characterize the susceptibility of *E. coli* isolates to disinfectants and heavy metals, propose ECOFFs, and investigate biofilm formation and resistance determinants to improve understanding of AMR ecology in tilapia aquaculture.

**Materials and Methods:**

A total of 333 *E. coli* isolates recovered from fish (n = 251) and cultivation water (n = 82) were investigated. Minimum inhibitory concentrations for five disinfectants and three heavy metals were determined using the agar dilution method, and ECOFFs were estimated using ECOFF Finder software. Biofilm formation was quantified using the tissue culture plate assay. We detected disinfectant- and heavy metal resistance genes by polymerase chain reaction. Principal component analysis was used to classify isolates according to resistance gene profiles, and multivariable logistic regression was performed to identify factors associated with the presence of the class 1 integron gene (*int1*).

**Results:**

A bimodal minimum inhibitory concentration distribution was observed for triclosan, supporting an ECOFF of 0.004 µg/mL; 52.6% of isolates exceeded this value, indicating a non-wild-type population with reduced susceptibility. In contrast, benzalkonium chloride, chlorhexidine (CHX), glutaraldehyde, and nitrilotriacetic acid exhibited unimodal distributions consistent with wild-type susceptibility. Among heavy metals, isolates were most susceptible to silver nitrate (minimum inhibitory concentration (MIC)₅₀ = 16 µg/mL), whereas higher MIC₅₀ values were observed for copper sulfate (CuSo_4_) and zinc chloride (ZnCl_2_). Most isolates (87.7%) exhibited weak biofilm formation, while strong biofilm producers were uncommon and predominantly recovered from cultivation water. Chromosomally encoded efflux pump genes, particularly *ydgE* (97.9%) and *mdfA* (92.8%), predominated, whereas plasmid-mediated disinfectant resistance genes were infrequent. Principal component analysis identified four distinct resistance gene clusters, and logistic regression demonstrated significant associations between *int1* and several antimicrobial and disinfectant resistance determinants.

**Conclusion:**

This study proposes the first ECOFFs for triclosan, selected disinfectants, and heavy metals in *E. coli* isolated from Thai tilapia farms and demonstrates the emergence of non-wild-type isolates with reduced triclosan susceptibility. The predominance of chromosomally encoded efflux pump genes and the observed resistance gene clustering suggest that intrinsic resistance mechanisms contribute substantially to bacterial persistence in aquaculture environments. These findings provide baseline evidence to support routine disinfectant susceptibility monitoring, prudent biocide use, and the integration of aquaculture into One Health AMR surveillance programs.

## INTRODUCTION

Aquaculture is one of the fastest-growing food production sectors and plays a vital role in meeting global demand for animal protein [1]. Tilapia is among the most widely cultivated fish species because of its rapid growth, adaptability to diverse environmental conditions, and high nutritional value [2]. In 2018, Thailand produced 216,600 tons of tilapia, accounting for nearly 4% of global production [3]. Despite the economic and food security importance of tilapia, the occurrence and dissemination of resistance to antimicrobials, disinfectants, and heavy metals in tilapia aquaculture systems remain insufficiently investigated, particularly in major producing countries such as Thailand and throughout Southeast Asia. Tilapia production commonly relies on open-water culture systems, including earthen ponds and floating cages, which are highly susceptible to contamination from municipal, agricultural, and industrial sources. Recent studies have documented a substantial burden of bacterial disease outbreaks in Thai tilapia aquaculture systems [4, 5]. As aquaculture continues to intensify under the combined pressures of climate change and deteriorating water quality, the risks of pathogen introduction, disease outbreaks, and contaminant accumulation increase, thereby threatening productivity, biosecurity, and sustainability [6].

Biocides are chemical agents used to control, inactivate, or eliminate harmful microorganisms. Increasing attention has focused on their environmental dissemination because these compounds exert selective pressure on microbial communities, thereby contributing to the emergence and persistence of antimicrobial resistance (AMR). Among these compounds, triclosan is a synthetic broad-spectrum antimicrobial agent widely incorporated into consumer products, including soaps, toothpaste, cosmetics, and household cleaning products. Because of its extensive use and incomplete removal during wastewater treatment, triclosan has become a major anthropogenic contaminant in aquatic ecosystems [7]. Other biocides have also been detected in environmental matrixes [8]. Benzalkonium chloride, a quaternary ammonium compound (QAC), is frequently detected in surface waters because of its widespread use in household cleaning products [9]. CHX, a bisbiguanide disinfectant widely used in clinical settings, serves as both a skin antiseptic and a surface disinfectant. Glutaraldehyde (GLU), a potent dialdehyde, is commonly used for the high-level disinfection of medical equipment and environmental surfaces. In contrast, nitrilotriacetic acid (NTA), although not an antimicrobial agent, functions as a chelating agent in detergents and industrial cleaning products. Following discharge into aquatic environments, these compounds may persist and contribute to AMR selection, thereby threatening the sustainability of aquaculture systems, particularly in regions with inadequate wastewater management.

Heavy metals such as copper (Cu) and zinc (Zn), widely used in aquaculture as dietary supplements to improve fish growth and immune function, also act as inorganic biocides that can select for AMR [10]. In Thailand, aquaculture standards require the use of approved disinfectants and chemicals according to manufacturers' instructions, together with the maintenance of usage records. Nevertheless, specific guidance and routine monitoring of cumulative exposure, environmental residues, and AMR-selective concentrations of disinfectants and heavy metals in tilapia farms remain limited. The subsequent accumulation of these compounds in aquatic environments raises significant concerns regarding their role in AMR selection and dissemination. Persistent environmental accumulation exerts continuous selective pressure on microbial communities, facilitating the development and maintenance of AMR, as demonstrated by the association between Cu exposure and increased resistance to macrolides and glycopeptides [11]. Although silver (Ag) is not routinely used in aquaculture, it enters aquatic environments through medical and industrial waste and may promote resistance because of its potent bactericidal activity [12]. Heavy metal resistance genes and AMR genes are frequently co-located on mobile genetic elements, facilitating horizontal gene transfer. Consequently, heavy metals function not only as environmental contaminants but also as important drivers of AMR in aquaculture ecosystems [13].

*Escherichia coli* is increasingly recognized as an important indicator organism and reservoir of AMR within aquaculture systems. Resistant strains have been isolated from fish intestines, pond water, and sediments and have been reported to harbor resistance genes against β-lactams, sulfonamides, quinolones, and aminoglycosides [14, 15]. Resistance to critically important antimicrobials, including colistin and extended-spectrum cephalon-sporins, has also been documented [16]. These resistance determinants are frequently associated with mobile genetic elements, including plasmids and integrons, which facilitate horizontal gene transfer and accelerate AMR dissemination within aquaculture systems [17, 18]. From a One Health perspective, the circulation of AMR *E. coli* and associated resistance determinants among aquatic animals, water, sediments, food products, and humans illustrates the close interconnection between animal, environmental, and human health. This interconnectedness contributes to AMR dissemination and poses a significant threat to public health and the sustainability of aquaculture production.

Cross-resistance between disinfectants and antimicrobials occurs when bacteria develop resistance to both classes of compounds. This phenomenon may result from shared mechanisms of action or common genetic adaptations, with multidrug efflux pumps among the principal mechanisms [19]. These pumps are encoded by both chromosomal and plasmid-borne genes [20]. Chromosomally encoded QAC resistance genes are prevalent in *E. coli* isolated from food-producing animals and farm environments where disinfectants are extensively used [21, 22]. Genes such as *mdfA* are associated with cross-resistance to tetracycline, chloramphenicol, norfloxacin, and benzalkonium chloride [23]. In addition, genes in the major facilitator superfamily (MFS), including *sugE(c)*, *emrE*, *ydgE*, and *ydgF*, encode membrane transporters that actively extrude toxic compounds and contribute to AMR [24]. Co-localization of AMR genes and disinfectant resistance genes has been reported in *E. coli* isolated from ready-to-eat meat in China, where all tested isolates were resistant to benzalkonium chloride, a QAC widely used in food production systems [25]. Similarly, co-resistance to carbapenems and disinfectants has been reported in *E. coli* isolated from fish in Vietnam [26]. Collectively, these findings highlight the public health concern posed by cross-resistance between antimicrobials and non-antibiotic biocides.

Although biocide-driven AMR has been documented in terrestrial livestock production systems, comparable evidence from aquaculture remains limited, particularly for tilapia production in Thailand. To date, no studies have proposed epidemiological cut-off values (ECOFFs) for disinfectants and heavy metals in *E. coli* isolated from Thai tilapia farms, nor have previous investigations integrated minimum inhibitory concentration (MIC) distribution analysis with biofilm formation, efflux pump gene profiling, and integron-associated resistance within a single study. This knowledge gap is particularly important because Thailand is one of the world's leading tilapia producers, where open-cage production systems may facilitate continuous exposure to environmental contaminants that select for AMR.

This study investigated the phenotypic and genotypic resistance of *E. coli* isolated from Thai tilapia aquaculture systems to disinfectants and heavy metals, evaluated biofilm-forming capacity, and examined the associations among resistance phenotypes, resistance determinants, and biofilm formation. We further analyzed MIC distributions to establish proposed ECOFFs for distinguishing putative wild-type and non-wild-type populations. To the best of our knowledge, this is the first comprehensive study to integrate MIC distribution analysis, proposed ECOFFs, biofilm phenotyping, and resistance gene clustering in *E. coli* from Thai tilapia aquaculture systems. The findings provide new insights into how environmental contaminants contribute to the development and dissemination of AMR and multidrug resistance in aquaculture and support evidence-based risk mitigation and sustainable aquaculture management from a One Health perspective.

## MATERIALS AND METHODS

### Ethical approval

The use, handling, storage, and laboratory investigation of *E. coli* isolates in this study were reviewed and approved by the Faculty of Veterinary Science Biosafety Committee (CU-VET-BC), Chulalongkorn University, Thailand, under Institutional Biosafety Committee (IBC) approval No. 2331030. This investigation used 333 previously collected *E. coli* isolates from the culture collection of the Department of Veterinary Public Health, Faculty of Veterinary Science, Chulalongkorn University, comprising isolates originating from fish (n = 251) and cultivation water (n = 82).

No animals were newly recruited, handled, sampled, or subjected to experimental procedures specifically for the present study. The bacterial isolates originated from samples collected in a previous study involving hybrid red tilapia raised in open-cage-culture systems. The original animal sampling protocol had been reviewed and approved by the Chulalongkorn University Animal Care and Use Committee (IACUC; Approval No. 2031048). Accordingly, the present study constituted laboratory-based analysis of archived bacterial isolates under the applicable institutional biosafety approval. All procedures involving bacterial cultures were conducted in accordance with the approved institutional biosafety requirements and laboratory procedures of Chulalongkorn University.

### Study period and location

This study was conducted from January to October 2021 at tilapia farms along the Kwae Noi River in Kanchanaburi, Thailand, 

### Study design and sources of isolates

A total of 333 *E. coli* isolates were obtained from the culture collection of the Department of Veterinary Public Health, Faculty of Veterinary Science, Chulalongkorn University. The isolates originated from two primary sources: fish samples (n = 251), comprising fish meat (n = 22), intestines (n = 42), liver and kidney (n = 99), and fish carcass rinses (n = 88), and cultivation water samples (n = 82). We collected samples between October 2019 and November 2020 from hybrid red tilapia raised in open-cage-culture systems. Farms were purposively selected based on production system, accessibility, and the availability of market-sized fish at the time of sampling. To minimize clonal duplication and ensure genomic diversity, we selected only one *E. coli* isolate from each sample, in accordance with the Food and Agriculture Organization guidelines for AMR surveillance in food animals [27]. The AMR profiles of these isolates have been reported previously using the agar dilution method [28].

### Phenotypic characterization of disinfectant resistance in *E. coli*

All isolates (n = 333) were evaluated for susceptibility to disinfectants and heavy metals by determining MICs using the agar dilution method with two-fold serial dilutions [29]. Because no standardized Clinical and Laboratory Standards Institute (CLSI) or European Committee on Antimicrobial Susceptibility Testing (EUCAST) methods or interpretive breakpoints are available for biocides and heavy metals, we adapted the procedure from the general MIC testing principles described in CLSI VET01, and selected concentration ranges based on previously published MIC distributions [29, 30].

Heavy metal stock solutions were prepared in sterile deionized water, completely dissolved, and inspected for precipitation before use. Each isolate was tested in triplicate. Five disinfectants commonly used as active ingredients in household products were evaluated: benzalkonium chloride (BKC; 0.5–1,024 µg/mL) (Sigma, St. Louis, MO, USA), CHX (0.5–1,024 µg/mL) (Sigma), GLU (256–4,096 µg/mL) (Sigma), nitrilotriacetic acid (NTA; 256–4,096 µg/mL) (Sigma), and triclosan (TCS; 0.002–8 µg/mL) (Sigma). Three heavy metals were also evaluated: AgNO₃ (0.5–128 µg/mL), CuSO₄ (256–4,096 µg/mL), and ZnCl₂ (256–4,096 µg/mL) (Sigma).

Reference strains included *Staphylococcus aureus* American Type Culture Collection (ATCC) 29213, *E. coli* ATCC 25922, and *Pseudomonas aeruginosa* ATCC 27853. We compared the MICs obtained for the quality-control strains with previously published reference MIC ranges to verify assay validity and reproducibility [30].

ECOFFs were determined for agents lacking established values in aquatic *E. coli* using ECOFF Finder software (version 2010-v2.1) [31], developed by EUCAST. We defined the ECOFF for each agent as the 97.5th percentile of the MIC distribution. MIC frequency data generated from the two-fold dilution series were entered into the software, which iteratively fitted a log-normal distribution to the presumed wild-type population. We evaluated the reliability of the 97.5th-percentile estimate by comparing the observed and modeled MIC distributions, and we rounded the calculated ECOFF up to the next tested two-fold dilution.

### Quantification of biofilm formation

All *E. coli* isolates were evaluated for biofilm formation using the tissue culture plate assay (TCPA) [32]. Pure cultures were revived on plate count agar (PCA; Difco), and two colonies from each isolate were inoculated into 3 mL of tryptic soy broth (TSB; Difco) and incubated at 37°C with shaking at 120 rpm for 16 ± 1 h. Cultures were adjusted to 1 × 10⁶ Colony-forming unit/mL, and 200 µL aliquots were transferred into sterile 96-well microplates and incubated statically at 37°C. Each isolate was tested in three technical replicates to ensure assay reproducibility. *E. coli* ATCC 25922 and sterile TSB served as the positive and negative controls, respectively.

Biofilm formation was quantified using the crystal violet (CV) assay [32]. After incubation, the wells were washed, air-dried for 4–6 h, fixed with 2% sodium acetate for 15 min, stained with 1% CV for 10 min, washed again, and air-dried. The bound stain was solubilized with 30% acetic acid, and the optical density (OD) was measured at 550 nm.

Biofilm production was classified according to the method described by Stepanović *et al.* [33] by comparing the OD of each isolate with the negative-control cut-off value (ODc):

1. OD ≤ ODc = No biofilm production

2. ODc < OD ≤ 2 × ODc = Weak biofilm production

3. 2 × ODc < OD ≤ 4 × ODc = Moderate biofilm production

4. OD > 4 × ODc = Strong biofilm production

### Genotypic characterization of disinfectant and heavy metal resistance genes

This study targeted disinfectant resistance genes associated with efflux pump mechanisms in *E. coli*, including chromosomally encoded genes (*sugE(c)*, *emrE*, *mdfA*, and *ydgE*) and the plasmid-encoded gene *sugE(p)*. In addition, we screened for small multidrug resistance family efflux pump genes (*qacE*, *qacEΔ1*, *qacF*, and *qacG*), which are frequently detected in Gram-negative bacteria and are associated with resistance to commonly used disinfectants. We also investigated five heavy metal resistance genes corresponding to the evaluated phenotypes, namely *silA*, *silE*, *copA*, *pcoA*, and *zntB* (Supplementary Table S1).

Genomic DNA was extracted using the whole-cell boiling method [34]. Polymerase chain reaction (PCR) was performed in a final reaction volume of 25 µL containing AllTaq DNA PCR Master Mix (Thermo Fisher Scientific, MA, USA), 5 µL of DNA template, 2 µL of each primer, and 16 µL of distilled water. PCR amplification consisted of an initial denaturation at 94°C for 5 min, followed by 30 amplification cycles, each comprising denaturation at 94°C for 30 s, annealing at the gene-specific temperature (Supplementary Table S1), and extension at 72°C for 30 s, followed by a final extension at 72°C for 7 min. PCR products were separated on 1.5% agarose gels stained with RedSafe™, visualized using an Omega Fluor™ imaging system (Aplegen), and compared with a 100-bp DNA ladder.

### Statistical analysis

Descriptive statistics were used to summarize biofilm production and the phenotypic and genotypic resistance profiles of the 333 *E. coli* isolates recovered from paired fish and water samples. Pearson's chi-square test was used to compare biofilm production between isolates recovered from fish and cultivation water.

Principal component analysis was performed to cluster isolates by resistance gene profiles. Multivariable logistic regression analysis was conducted to identify factors associated with the presence of *int1*, a marker of classical and non-classical integron-mediated multidrug resistance. Predictor variables included AMR, disinfectant resistance, and biofilm formation level. We selected candidate variables based on biological relevance and significant univariable associations. We evaluated multicollinearity using variance inflation factors and assessed model performance using the Hosmer-Lemeshow goodness-of-fit test. Statistical significance was defined as p < 0.05. All statistical analyses were performed using Stata version 18.0 (StataCorp, College Station, TX, USA).

## RESULTS

### Phenotypic characterization of disinfectant and heavy metal resistance in *E. coli*

The MIC distributions and proposed ECOFFs are presented in Table 1. A bimodal TCS MIC distribution was observed among tilapia-derived *E. coli* isolates, particularly those recovered from cultivation water, enabling the proposal of an ECOFF of 0.004 µg/mL. In contrast, the other disinfectants exhibited unimodal MIC distributions (Supplementary Figure S1). BKC MICs ranged from 4 to 256 µg/mL in isolates from both sources, whereas CHX MICs were predominantly 2–32 µg/mL in fish isolates and 4–16 µg/mL in water isolates. GLU and nitrilotriacetic acid (NTA) exhibited high MICs ranging from 1,024 µg/mL to >4,096 µg/mL.

All tested heavy metals exhibited narrow MIC distributions. The lowest MIC₅₀ was observed for AgNO₃ (16 µg/mL), whereas higher MIC₅₀ values were recorded for CuSO₄ (2,048 µg/mL) and ZnCl₂ (1,024 µg/mL). Overall, the MIC distributions were similar between fish and water isolates, with a significant difference observed only for AgNO₃.

### Quantification of biofilm formation

Most *E. coli* isolates (87.7%) exhibited weak biofilm production (Table 2). Strong biofilm production was uncommon (4.5%) and was detected predominantly among isolates recovered from cultivation water (2.4%), followed by intestinal samples (1.5%) and fish carcass rinses (0.6%). Biofilm production differed significantly between isolates recovered from fish and cultivation water (χ² = 5.9, p = 0.015). Isolates recovered from cultivation water had approximately twice the odds of biofilm formation compared with those recovered from fish (Odds ratio [OR] = 1.89; 95% CI = 1.13–3.16).

### Genotypic characterization of disinfectant and heavy metal resistance genes in *E. coli*

**Figure 1:**
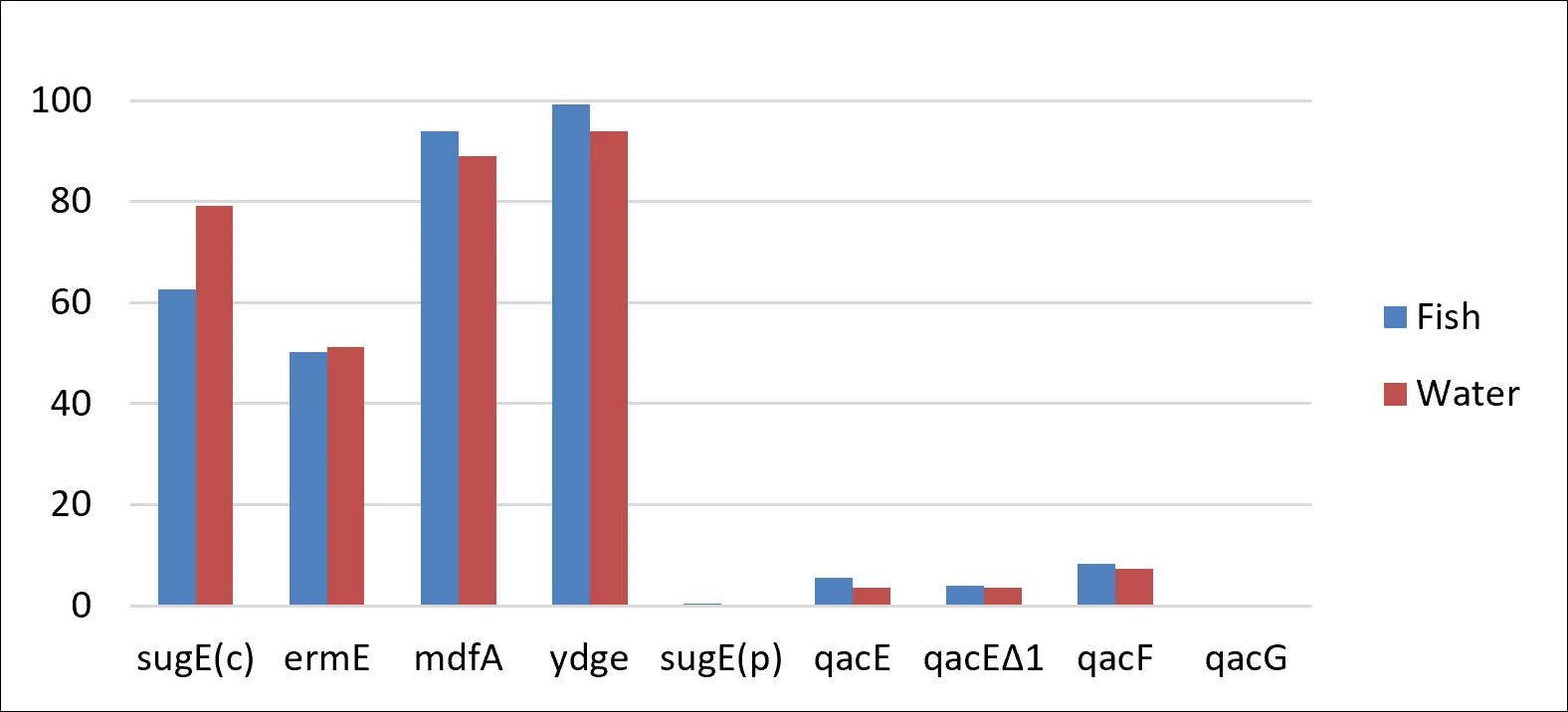
Occurrence of disinfectant resistance genes in *Escherichia coli* isolates (n = 333).

The most frequently detected disinfectant resistance genes were *ydgE* (97.9%), followed by *mdfA* (92.8%), *sugE(c)* (66.7%), and *emrE* (50.5%) (Figure 1). In contrast, *qacF* (8.1%), *qacE* (5.1%), and *qacEΔ1* (3.9%) were detected at low frequencies, whereas *qacG* was not detected. None of the five tested heavy metal resistance genes were detected in the *E. coli* isolates. Disinfectant and heavy metal resistance genes previously reported in *E. coli* from aquaculture and terrestrial production systems are summarized in Supplementary Table S2.

**Table 1:** Minimum inhibitory concentrations of disinfectants and heavy metals in E. coli isolates recovered from fish (n = 251) and cultivation water (n = 82).

**Biocide**	**Source**	**0.002**	**0.004**	**0.008**	**0.016**	**0.031**	**0.063**	**0.125**	**0.25**	**0.5**	**1**	**2**	**4**	**8**	**16**	**32**	**64**	**128**	**256**	**512**	**1,024**	**2,048**	**4,096**	**>4,096**	**MIC₅₀**	**Proposed ECOFF**
TCS	Fish	106	20	0	0	4	6	11	52	35	17	–	–	–	–	–	–	–	–	–	–	–	–	–	0.004	0.004
	Water	30	2	0	0	1	3	12	11	10	13	–	–	–	–	–	–	–	–	–	–	–	–	–	0.125	0.004
BKC	Fish	–	–	–	–	–	–	–	–	–	–	–	2	0	1	105	113	18	12	–	–	–	–	–	64	128
	Water	–	–	–	–	–	–	–	–	–	–	–	–	–	1	38	35	6	2	–	–	–	–	–	64	64
CHX	Fish	–	–	–	–	–	–	–	–	–	–	3	212	35	0	1	–	–	–	–	–	–	–	–	4	16
	Water	–	–	–	–	–	–	–	–	–	–	–	73	8	1	–	–	–	–	–	–	–	–	–	4	16
GLU	Fish	–	–	–	–	–	–	–	–	–	–		–	–	–	–	–	–	–	–	–	3	211	37	4,096	4,096
	Water	–	–	–	–	–	–	–	–	–	–		–	–	–	–	–	–	–	–	–	1	74	7	4,096	4,096
NTA	Fish	–	–	–	–	–	–	–	–	–	–		–	–	–	–	–	–	–		30	0	37	184	>4,096	>4,096
	Water	–	–	–	–	–	–	–	–	–	–		–	–	–	–	–	–	–	–	12	0	7	63	>4,096	>4,096
AgNO₃	Fish	–	–	–	–	–	–	–	–	–	–		–	–	171	80	–	–	–	–	–	–	–	–	16	32
	Water	–	–	–	–	–	–	–	–	–	–		–	–	45	37	–	–	–	–	–	–	–	–	16	32
CuSO₄	Fish	–	–	–	–	–	–	–	–	–	–		–	–	–	–	–	–	–	–	1	250	–	–	2,048	2,048
	Water	–	–	–	–	–	–	–	–	–	–		–	–	–	–	–	–		–	–	82	–	–	2,048	2,048
ZnCl₂	Fish	–	–	–	–	–	–	–	–	–	–		–	–	–	–	–	–		3	187	61	–	–	1,024	2,048
	Water	–	–	–	–	–	–	–	–	–	–		–	–	–	–	–	–		1	56	25	–	–	1,024	2,048

AgNO₃ = Silver nitrate; BKC = Benzalkonium chloride; CHX = Chlorhexidine; CuSO₄ = Copper sulfate; ECOFF = Epidemiological cut-off value; GLU = Glutaraldehyde; MIC = Minimum inhibitory concentration; MIC₅₀ = Minimum inhibitory concentration inhibiting 50% of isolates; NTA = Nitrilotriacetic acid; TCS = Triclosan; ZnCl₂ = Zinc chloride.

**Table 2:** Distribution of biofilm production levels among Escherichia coli isolates (n = 333).

**Sample type**	**Weak**	**Moderate**	**Strong**
Fish meat (n = 22)	22 (6.6%)	0 (0.0%)	0 (0.0%)
Intestine (n = 100)	87 (26.1%)	8 (2.4%)	5 (1.5%)
Liver and kidney (n = 42)	40 (12.0%)	2 (0.6%)	0 (0.0%)
Fish carcass rinse (n = 88)	79 (23.7%)	7 (2.1%)	2 (0.6%)
Cultivation water (n = 81)	64 (19.2%)	9 (2.7%)	8 (2.4%)
Total	292 (87.7%)	26 (7.8%)	15 (4.5%)

### Cluster analysis and multivariable logistic regression analysis

**Figure 2:**
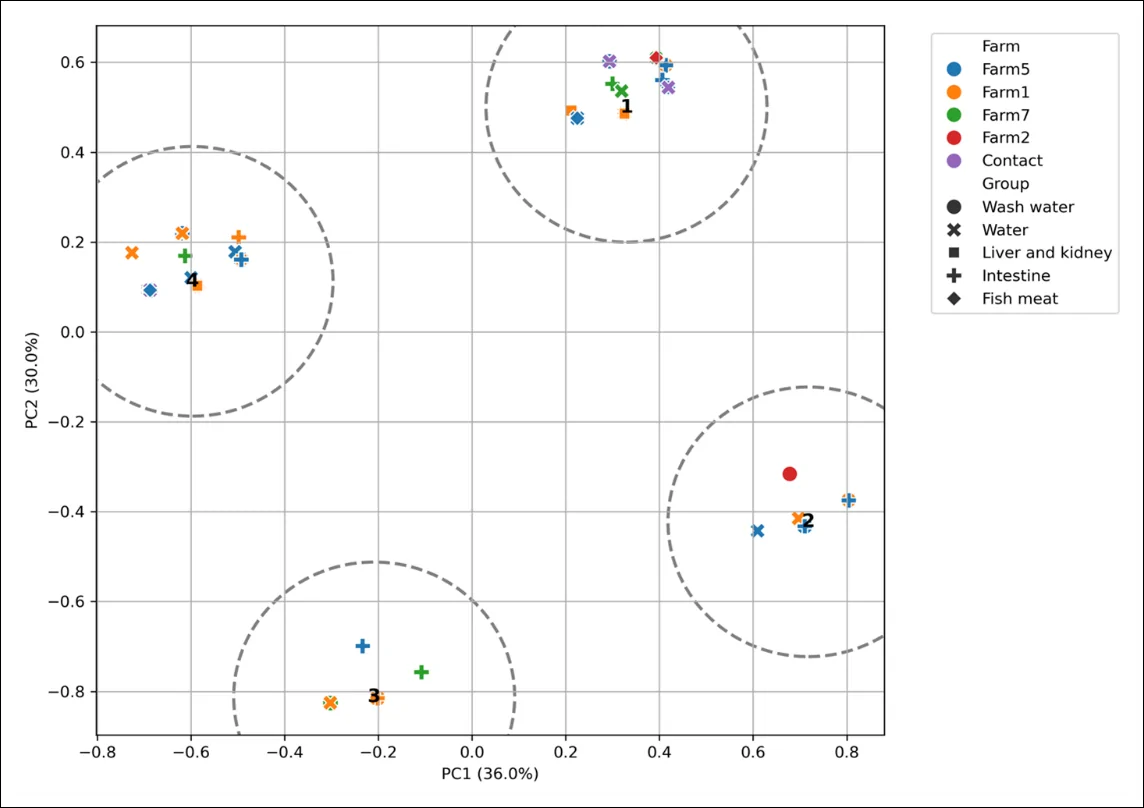
Principal component analysis plot showing clustering of *Escherichia coli* isolates from tilapia aquaculture based on the presence or absence of disinfectant and heavy metal resistance genes.

Clustering based on the presence or absence of resistance genes identified four distinct clusters (Figure 2). Each cluster contained isolates from different sample types and farm origins but differed in its predominant resistance gene profile (Supplementary Table S3). Clusters 1 and 2 were predominantly characterized by *emrE*, whereas Clusters 3 and 4 were predominantly characterized by *ydgE* and *sugE(c)*. The plasmid-encoded genes *qacE*, *qacEΔ1*, and *qacF* were detected across all clusters. Notably, *sugE(p)* was detected exclusively in Cluster 4. 

Multivariable logistic regression analysis showed that the presence of *int1* was significantly and positively associated with *strA*, *tetA*, and *aadA2*, whereas it was significantly and negatively associated with *floR*. The presence of *int1* was also significantly associated with the disinfectant resistance genes *qacF*, *emrE*, and *mdfA* (Table 3).

**Table 3:** Multivariable logistic regression analysis of antimicrobial and disinfectant resistance gene predictors associated with the presence of int1 in Escherichia coli from tilapia aquaculture.

**Predictor**	**Odds ratio**	**Standard errorᵃ**	**CI**	**p-value**
*strA*	80.37	1.96	3.06–5.71	<0.0001
*tetA*	7.89	1.57	1.18–2.95	<0.0001
*floR*	0.11	2.00	−3.58–−0.87	0.001
*aadA2*	17.48	1.86	1.65–4.08	<0.0001
*qacF*	14.13	2.07	1.22–4.07	<0.0001
*emrE*	3.11	1.60	0.22–2.05	0.016
*mdfA*	16.16	3.30	0.44–5.12	0.020
Constant	0.00075	4.02	−9.92–−4.46	<0.0001

Akaike information criterion = 162.43. ᵃStandard error. CI = Confidence interval.

## DISCUSSION

### Phenotypic resistance profiles to disinfectants and heavy metals in *E. coli*

This study provides a comprehensive assessment of AMR in *E. coli* isolated from tilapia aquaculture, focusing on susceptibility to commonly used disinfectants and heavy metals, together with biofilm-forming capacity. Five disinfectants commonly used in household and environmental settings were selected based on previous evidence [7]. A bimodal TCS MIC distribution was observed, indicating two distinct bacterial subpopulations: wild-type isolates with intrinsic susceptibility and non-wild-type isolates with acquired reduced susceptibility or tolerance to TCS. Notably, some isolates had MICs exceeding 1 µg/mL, higher than those previously reported for *E. coli* isolated from animal production systems in Thailand [30].

The bimodal MIC distribution, along with MIC values exceeding 1 µg/mL, supports the presence of a distinct subpopulation with reduced susceptibility to TCS and indicates a shift in the susceptibility profile of *E. coli* in tilapia aquaculture. TCS inhibits bacterial fatty acid biosynthesis by targeting the enoyl-acyl carrier protein reductase FabI, encoded by *fabI*, and mutations in *fabI* represent one of the principal mechanisms underlying TCS resistance [35]. In addition, increased activity of multidrug efflux pumps, particularly resistance-nodulation-division systems such as AcrAB-TolC and MexAB-OprM, may contribute to reduced intracellular accumulation of TCS and thereby promote reduced susceptibility [36]. The MIC₅₀ values of the remaining disinfectants observed in this study were slightly higher than those previously reported for *E. coli* isolated from animal food production systems (Table 4) [30, 37–41], suggesting reduced susceptibility and the potential emergence of tolerance to multiple disinfectants.

Compared with the only previous study conducted in Thailand using isolates from the swine production chain, the MIC₅₀ values for BKC, CHX, GLU, and TCS were at least one two-fold dilution higher [30]. This shift in susceptibility has important implications for farm biosecurity because disinfectant efficacy is influenced by water quality, temperature, disinfectant concentration, contact time, and organic matter [42]. By providing susceptibility data from Thai tilapia aquaculture, the present study offers region-specific evidence supporting thorough pre-cleaning procedures and the application of disinfectants at validated concentrations with adequate contact times to maximize bacterial inactivation while minimizing exposure to sublethal concentrations.

Differences between the proposed ECOFFs in the present study and those reported previously may reflect variations in the ecological origin of the isolates, local patterns of disinfectant use, and sample size. Nevertheless, these ECOFFs provide a valuable baseline for monitoring temporal changes in susceptibility and detecting the emergence of non-wild-type populations during aquaculture surveillance. The observed differences may also be associated with the prolonged environmental persistence of disinfectants in aquaculture systems. For example, BKC has been reported to persist in water for up to 38 days, thereby maintaining continuous selective pressure on microbial communities [43]. Collectively, these findings highlight the role of disinfectants in driving AMR in aquaculture through sustained environmental selective pressure, thereby facilitating the emergence and persistence of resistant bacterial populations.

**Table 4:** Reported MIC₅₀ values of biocides against Escherichia coli isolated from animal and environmental sources.

**Biocide**	**Source**	**n**	**MIC₅₀ (µg/mL)**	**Reference**
BKC	Pig and retail meats	32	32	[37]
	Chicken and farms	34	8	[38]
	Pig, pork, and carcass	864	32	[30]
	Pig and pork	199	32	[39]
	Slaughterhouses	75	32	[40]
	Slaughterhouses	50	16	[41]
CHX	Chicken and farms	34	0.5	[38]
	Pig, pork, and carcass	864	2	[30]
	Pig and pork	199	1-2	[39]
	Slaughterhouses	75	1	[40]
	Slaughterhouses	50	1	[41]
GLU	Pig, pork, and carcass	864	2,048	[30]
	Pig and pork	199	512	[39]
	Slaughterhouses	75	2,048	[40]
TCS	Pig, pork, and carcass	864	0.03125	[30]
	Slaughterhouses	50	1	[41]

BKC = Benzalkonium chloride; CHX = Chlorhexidine; GLU = Glutaraldehyde; MIC₅₀ = Minimum inhibitory concentration inhibiting 50% of isolates; TCS = Triclosan.

All tested heavy metals exhibited limited variation in MIC distributions, suggesting relatively uniform selective pressure within the studied aquaculture environment. The MIC₅₀ of AgNO₃ (16 µg/mL) observed in the present study was higher than that previously reported for *E. coli* isolated from tilapia in Zimbabwe, possibly reflecting adaptation to anthropogenic silver-containing biocides in Thai aquaculture systems [44]. Likewise, the MIC₅₀ values for CuSO₄ (2,048 µg/mL) and ZnCl₂ (1,024 µg/mL) exceeded those reported for *E. coli* isolated from fishponds in Nigeria [45]. These findings raise concerns regarding inappropriate concentrations of heavy metals in commercial fish feeds, which may contribute to environmental accumulation and promote AMR [46]. The limited regulatory oversight of heavy metal use may further increase the ecological and public health risks associated with these environmental contaminants.

### Biofilm formation of *E. coli*

Strong biofilm production was generally uncommon, indicating limited biofilm-forming potential among *E. coli* isolates recovered from tilapia aquaculture. Nevertheless, a small subpopulation of strong biofilm-producing isolates was identified, consistent with previous findings in fish from Vietnam [47]. Biofilms protect bacteria against disinfectants and antimicrobials, thereby facilitating the persistence of AMR and multidrug-resistant (MDR) strains [48]. Previous studies have also demonstrated a positive association between biofilm formation and resistance to QACs [49]. Collectively, these findings highlight the importance of implementing targeted strategies to mitigate biofilm-mediated resistance and reduce its environmental and public health impacts.

### Genotypic resistance profiles to disinfectants and heavy metals in *E. coli*

The *E. coli* isolates in the present study lacked all five targeted heavy metal resistance genes encoding metal efflux pumps, consistent with the very low prevalence of these genes reported in bacteria isolated from fish-cage sediments in Turkey [50]. This finding suggests that heavy metal tolerance in these aquaculture environments may be mediated through alternative resistance mechanisms. Resistance to QACs was primarily associated with chromosomally encoded efflux pump genes. Among the disinfectant resistance genes investigated, *ydgE* and *mdfA* were the most prevalent, consistent with findings reported for *E. coli* isolated from retail fish in India [44]. The *mdfA* gene confers cross-resistance by exporting both tetracyclines and BKC, thereby reducing the effectiveness of both antimicrobials and disinfectants [20]. Other chromosomally encoded genes, including *sugE(c)* and *emrE*, which are associated with high-level resistance to QACs, were also detected. In contrast, the prevalence of plasmid-associated QAC resistance genes, including *qacE*, *qacEΔ1*, *qacF*, *qacG*, and *sugE(p)*, was very low. These findings suggest that chromosomally encoded mechanisms may play a greater role than plasmid-mediated mechanisms in disinfectant resistance among *E. coli* isolates from tilapia aquaculture.

Despite the observed heavy metal tolerance, none of the targeted heavy metal resistance genes were detected. This discrepancy suggests that the observed phenotype may result from intrinsic metal homeostasis, regulatory co-expression, cross-resistance involving other efflux systems, or additional resistance determinants that were not investigated in the present study [51]. These findings emphasize the importance of monitoring both intrinsic and acquired resistance mechanisms in aquatic environments.

### Clustering of resistance genes

Cluster analysis demonstrated that the chromosomally encoded genes *ydgE*, *sugE(c)*, and *emrE* predominated within specific clusters, although each cluster remained heterogeneous with respect to sample type, farm origin, and overall resistance gene profile. The predominance of these genes within individual clusters suggests possible co-selection under environmental pressures, such as repeated exposure to disinfectants. In contrast, the plasmid-associated genes *qacE*, *qacEΔ1*, and *qacF* were detected across all clusters, suggesting widespread dissemination that may be facilitated through horizontal gene transfer (HGT), as reported in a previous study [52]. The co-occurrence of chromosomally encoded efflux pump genes and plasmid-mediated resistance determinants further highlights the interaction between intrinsic and acquired mechanisms in shaping disinfectant resistance profiles. Furthermore, the heterogeneous clustering patterns may reflect multiple contamination sources and diverse evolutionary pathways of resistance acquisition within aquaculture systems. Collectively, these findings illustrate the complexity of resistance gene distribution and emphasize the influence of environmental selective pressures on the maintenance and dissemination of antimicrobial and biocide resistance in aquatic ecosystems.

### Multivariable analysis of integron-associated biocide and AMR gene co-occurrence

Multivariable logistic regression analysis provided novel insights into the co-occurrence of resistance determinants in *E. coli* isolated from Thai tilapia aquaculture. The presence of *int1* was significantly associated with *strA*, *tetA*, *aadA2*, *qacF*, *emrE*, and *mdfA*. These associations indicate that *int1*-positive isolates may harbor broader and more diverse resistance profiles, reflecting the combined effects of mobile genetic elements, genetic linkage, and co-selection by antimicrobials and biocides. Classical integrons typically contain *qacEΔ1* and *sul1*, whereas non-classical integrons may harbor alternative genes, including *qacH* and *sul3* [53]. However, the multivariable analysis did not support either of these classical integron configurations. This finding suggests that alternative mechanisms, including plasmid-mediated transfer, transposons, and co-selection driven by disinfectants and heavy metals, may contribute to the dissemination of disinfectant resistance genes in aquatic environments. From a One Health perspective, simultaneous monitoring of *int1* alongside antimicrobial and biocide resistance genes in fish, farm water, sediments, and surrounding aquatic environments could improve early detection and surveillance of resistance dissemination across environmental, animal, and human interfaces.

### Novel contributions and significance of the present study

This study provides the first evidence of reduced TCS susceptibility among *E. coli* isolates recovered from Thai tilapia aquaculture and proposes the first ECOFFs for multiple disinfectants and heavy metals in this production system. Unlike previous investigations focused primarily on terrestrial livestock, this study integrates MIC distributions, biofilm-forming capacity, and resistance gene profiling to provide a comprehensive assessment of biocide susceptibility in an aquatic production environment. The predominance of chromosomally encoded resistance determinants over plasmid-mediated mechanisms suggests that intrinsic resistance mechanisms play a major role in disinfectant tolerance in these isolates. The identification of non-wild-type populations with reduced susceptibility to TCS, reflected by a bimodal MIC distribution, is an important finding indicating a shift in susceptibility under natural aquaculture conditions. Such reduced susceptibility raises concerns regarding the long-term effectiveness of commonly used disinfectants and the potential for co-selection of AMR. Although HGT likely contributes to resistance dissemination, the present findings suggest that its role is secondary to intrinsic chromosomal mechanisms. Furthermore, the observed resistance gene clustering indicates potential selection under prolonged disinfectant exposure, whereas the presence of strong biofilm-forming isolates suggests that biofilms may serve as protective niches that facilitate the persistence and dissemination of resistant bacteria in aquaculture environments.

### Limitations and future research directions

Several limitations should be considered when interpreting the findings of this study. First, the isolates were obtained from a limited geographic area and a relatively small number of tilapia farms, which may limit the generalizability of the findings to other aquaculture production systems. Second, the targeted PCR approach focused on selected disinfectant and heavy metal resistance genes and therefore may not have identified additional resistance mechanisms involved in reduced susceptibility. Third, environmental concentrations of disinfectants, biocides, and heavy metals were not quantified, precluding direct assessment of the relationship between environmental exposure and the observed MIC distributions. Furthermore, the cross-sectional sampling design did not permit evaluation of temporal changes in susceptibility or the emergence of non-wild-type populations. Finally, clonal relatedness among isolates was not investigated; therefore, it was not possible to determine whether similar resistance profiles resulted from clonal dissemination or the independent acquisition of resistance determinants.

Despite these limitations, this study provides valuable baseline data on disinfectant and heavy metal susceptibility in *E. coli* from Thai tilapia aquaculture and establishes proposed ECOFFs for future surveillance. Future investigations should incorporate longitudinal and multicenter sampling, quantify environmental concentrations of disinfectants and heavy metals, and employ whole-genome sequencing and comparative genomic analyses to characterize resistance mechanisms, mobile genetic elements, and transmission pathways. Integrating environmental monitoring with phenotypic and genomic surveillance will improve understanding of the drivers of disinfectant tolerance and AMR dissemination and will support evidence-based risk assessment and sustainable aquaculture management within a One Health framework.

## CONCLUSION

This study provides the first comprehensive assessment of disinfectant and heavy metal susceptibility in *E. coli* isolated from Thai tilapia aquaculture and establishes the first proposed ECOFFs for multiple disinfectants and heavy metals in this production system. A bimodal TCS MIC distribution identified a distinct non-wild-type population with reduced susceptibility, whereas BKC, CHX, GLU, and NTA exhibited unimodal MIC distributions consistent with predominantly wild-type populations. Most isolates exhibited weak biofilm-forming capacity, while disinfectant resistance was primarily associated with chromosomally encoded efflux pump genes, particularly *ydgE* and *mdfA*, with a low prevalence of plasmid-mediated QAC resistance genes. Cluster analysis and multivariable logistic regression further demonstrated the co-occurrence of AMR and disinfectant resistance determinants, highlighting the contribution of intrinsic resistance mechanisms and potential co-selection within tilapia aquaculture environments.

The proposed ECOFFs provide a practical framework for distinguishing wild-type and non-wild-type populations during routine surveillance of disinfectant susceptibility in aquaculture. The observed shift toward reduced TCS susceptibility emphasizes the importance of evidence-based disinfectant selection, appropriate working concentrations, adequate contact times, and regular evaluation of disinfectant efficacy. Incorporating disinfectant susceptibility monitoring together with AMR surveillance will strengthen biosecurity programs, support prudent disinfectant use, and contribute to sustainable tilapia production within a One Health framework.

A major strength of this study is the integration of phenotypic susceptibility testing, MIC distribution analysis, proposed ECOFFs, biofilm phenotyping, resistance gene profiling, cluster analysis, and multivariable statistical analysis using a relatively large collection of *E. coli* isolates recovered from paired fish and cultivation water samples. This integrated approach provides a comprehensive understanding of disinfectant and heavy metal susceptibility and the potential mechanisms contributing to resistance persistence in aquaculture environments.

Overall, this study demonstrates that reduced susceptibility to disinfectants, particularly TCS, has emerged among *E. coli* isolated from Thai tilapia aquaculture and that chromosomally encoded resistance mechanisms predominate over plasmid-mediated determinants. The proposed ECOFFs provide an important baseline for future surveillance and facilitate standardized interpretation of disinfectant susceptibility. These findings support the integration of disinfectant susceptibility monitoring into routine AMR surveillance and reinforce the need for coordinated One Health strategies to reduce selective pressure, improve biosecurity, and mitigate the dissemination of resistant bacteria across aquaculture, environmental, animal, and human health sectors.

## DATA AVAILABILITY

The supplementary data can be made available from the corresponding author upon request.

## GENERATIVE AI DECLARATION

The authors declare that generative artificial intelligence (AI) tools were used solely to improve language, grammar, and readability during manuscript preparation. All scientific content, data analysis, interpretation of results, and conclusions were developed and verified by the authors. The authors take full responsibility for the accuracy, integrity, and originality of the work presented, and no AI tool was listed as an author.

## AUTHORS’ CONTRIBUTIONS

VT: Conceptualization, methodology, investigation, data curation, formal analysis, data interpretation, project administration, visualization, writing of the original draft, and writing—review and editing. JS: Conceptualization, methodology, investigation, data curation, formal analysis, data interpretation, and writing—review and editing. SA: Methodology, investigation, data curation, and manuscript review. NR: Methodology and manuscript review. WH: Methodology, formal analysis, data interpretation, and writing—review and editing. SJ: Conceptualization, methodology, formal analysis, data interpretation, funding acquisition, supervision, and writing—review and editing. All authors have read and approved the final manuscript.

## References

[1] Tigchelaar M, Leape J, Micheli F, Allison EH, Basurto X, Bennett A (2022). The vital roles of blue foods in the global food system. Glob Food Secur.

[2] Garlock T, Asche F, Anderson JL, Hilsenroth J, Lorenzen K, Pincinato RBM (2023). Global and regional determinants of diversity in blue foods. Rev Fish Sci Aquac.

[3] Miao W, Wang W (2020). Trends of aquaculture production and trade: carp, tilapia, and shrimp. Asian Fish Sci.

[4] Vinarukwong N, Lukkana M, Berntsen JO, Wongtavatchai J (2018). Therapeutic use of sulfadimethoxine-ormetoprim for control of Streptococcus agalactiae infection in Nile tilapia (Oreochromis niloticus) fry. Thai J Vet Med.

[5] Thaotumpitak V, Sripradite J, Atwill ER, Tepaamorndech S, Jeamsripong S (2022). Bacterial pathogens and factors associated with Salmonella contamination in hybrid red tilapia (Oreochromis spp.) cultivated in a cage culture system. Food Qual Saf.

[6] Cascarano MC, Stavrakidis-Zachou O, Mladineo I, Thompson KD, Papandroulakis N, Katharios P (2021). Mediterranean aquaculture in a changing climate: temperature effects on pathogens and diseases of three farmed fish species. Pathogens.

[7] Jabłońska‐Trypuć A (2023). A review on triclosan in wastewater: mechanism of action, resistance phenomenon, environmental risks, and sustainable removal techniques. Water Environ Res.

[8] Basiry D, Entezari Heravi NE, Uluseker C, Kaster KM, Kommedal R, Pala-Ozkok I (2022). The effect of disinfectants and antiseptics on co- and cross-selection of resistance to antibiotics in aquatic environments and wastewater treatment plants. Front Microbiol.

[9] Pati SG, Arnold WA (2020). Comprehensive screening of quaternary ammonium surfactants and ionic liquids in wastewater effluents and lake sediments. Environ Sci Process Impacts.

[10] Dawood MAO (2022). Dietary copper requirements for aquatic animals: a review. Biol Trace Elem Res.

[11] Hasman H, Kempf I, Chidaine B, Cariolet R, Ersbøll AK, Houe H (2006). Copper resistance in Enterococcus faecium, mediated by the tcrB gene, is selected by supplementation of pig feed with copper sulfate. Appl Environ Microbiol.

[12] Li H, Xu H (2024). Mechanisms of bacterial resistance to environmental silver and antimicrobial strategies for silver: a review. Environ Res.

[13] Souza SSR, Turcotte MR, Li J, Zhang X, Wolfe KL, Gao F (2022). Population analysis of heavy metal and biocide resistance genes in Salmonella enterica from human clinical cases in New Hampshire, United States. Front Microbiol.

[14] Hoa TTT, Nakayama T, Huyen HM, Harada K, Hinenoya A, Phuong NT (2020). Extended‐spectrum beta-lactamase-producing Escherichia coli harbouring sul and mcr-1 genes isolates from fish gut contents in the Mekong Delta, Vietnam. Lett Appl Microbiol.

[15] Sivaraman GK, Sudha S, Muneeb KH, Shome B, Holmes M, Cole J (2020). Molecular assessment of antimicrobial resistance and virulence in multi drug resistant ESBL-producing Escherichia coli and Klebsiella pneumoniae from food fishes, Assam, India. Microb Pathog.

[16] Nakayama T, Hoa TTT, Huyen HM, Yamaguchi T, Jinnai M, Minh DTN (2022). Isolation of carbapenem-resistant Enterobacteriaceae harbouring NDM-1, 4, 5, OXA48 and KPC from river fish in Vietnam. Food Control.

[17] Zhu L, Zhou Z, Liu Y, Lin Z, Shuai X, Xu L (2020). Comprehensive understanding of the plasmid-mediated colistin resistance gene mcr-1 in aquatic environments. Environ Sci Technol.

[18] Singh NS, Singhal N, Virdi JS (2018). Genetic environment of blaTEM-1, blaCTX-M-15, blaCMY-42 and characterization of integrons of Escherichia coli isolated from an Indian urban aquatic environment. Front Microbiol.

[19] Merchel Piovesan Pereira B, Wang X, Tagkopoulos I (2021). Biocide-induced emergence of antibiotic resistance in Escherichia coli. Front Microbiol.

[20] Zou L, Meng J, McDermott PF, Wang F, Yang Q, Cao G (2014). Presence of disinfectant resistance genes in Escherichia coli isolated from retail meats in the USA. J Antimicrob Chemother.

[21] Enany ME, Algammal AM, Nasef SA, Abo-Eillil SAM, Bin-Jumah M, Taha AE (2019). The occurrence of the multidrug resistance (MDR) and the prevalence of virulence genes and QACs resistance genes in E. coli isolated from environmental and avian sources. AMB Express.

[22] Sahin S, Mogulkoc MN, Kürekci C (2022). Disinfectant and heavy metal resistance profiles in extended spectrum β-lactamase (ESBL) producing Escherichia coli isolates from chicken meat samples. Int J Food Microbiol.

[23] Vilela FP, Rodrigues DDP, Allard MW, Falcão JP (2022). Prevalence of efflux pump and heavy metal tolerance encoding genes among Salmonella enterica serovar Infantis strains from diverse sources in Brazil. PLoS One.

[24] Wan Y, Wang M, Chan EWC, Chen S (2021). Membrane transporters of the major facilitator superfamily are essential for long-term maintenance of phenotypic tolerance to multiple antibiotics in E. coli. Microbiol Spectr.

[25] Li L, Ye L, Kromann S, Meng H (2017). Occurrence of extended-spectrum β-lactamases, plasmid-mediated quinolone resistance, and disinfectant resistance genes in Escherichia coli isolated from ready-to-eat meat products. Foodborne Pathog Dis.

[26] Nakayama T, Yamaguchi T, Minh DTN, Hoang ON, Thi HL, Thanh PN (2023). Carbapenem-resistant Citrobacter freundii and Escherichia coli harboring a common IncC-Type plasmid encoding IS26 upstream of blaNDM-1, sul1, aph(3′)-VI, and qacE isolated from edible Mastacembelidae fish. Microbiol Resour Announc.

[27] Food and Agriculture Organization of the United Nations (2019). Monitoring and surveillance of antimicrobial resistance in bacteria from healthy food animals intended for consumption: regional antimicrobial resistance monitoring and surveillance guidelines.

[28] Thaotumpitak V, Tepaamorndech S, Sripradite J, Atwill ER, Anuntawirun S, Chantarasakha K (2024). Insights into antimicrobial and multidrug resistance of Escherichia coli in hybrid red tilapia cultivation. Aquaculture.

[29] Clinical and Laboratory Standards Institute (2015). Performance standards for antimicrobial disk and dilution susceptibility tests for bacteria isolated from animals CLSI supplement VET01-S3.

[30] Puangseree J, Jeamsripong S, Prathan R, Pungpian C, Chuanchuen R (2021). Resistance to widely-used disinfectants and heavy metals and cross resistance to antibiotics in Escherichia coli isolated from pigs, pork and pig carcass. Food Control.

[31] Turnidge J, Kahlmeter G, Kronvall G (2006). Statistical characterisation of bacterial wild‐type MIC value distributions and the determination of epidemiological cut‐off values. Clin Microbiol Infect.

[32] Peeters E, Nelis HJ, Coenye T (2008). Comparison of multiple methods for quantification of microbial biofilms grown in microtiter plates. J Microbiol Methods.

[33] Stepanović S, Vuković D, Dakić I, Savić B, Švabić-Vlahović M (2000). A modified microtiter-plate test for quantification of staphylococcal biofilm formation. J Microbiol Methods.

[34] Lévesque C, Piche L, Larose C, Roy PH (1995). PCR mapping of integrons reveals several novel combinations of resistance genes. Antimicrob Agents Chemother.

[35] Khan R, Roy N, Choi K, Lee SW (2018). Distribution of triclosan-resistant genes in major pathogenic microorganisms revealed by metagenome and genome-wide analysis. PLoS One.

[36] Shrestha P, Zhang Y, Chen WJ, Wong TY (2020). Triclosan: antimicrobial mechanisms, antibiotics interactions, clinical applications, and human health. J Environ Sci Health C.

[37] Wu G, Yang Q, Long M, Guo L, Li B, Meng Y (2015). Evaluation of agar dilution and broth microdilution methods to determine the disinfectant susceptibility. J Antibiot.

[38] Deus D, Krischek C, Pfeifer Y, Sharifi AR, Fiegen U, Reich F (2017). Comparative analysis of the susceptibility to biocides and heavy metals of extended-spectrum β-lactamase-producing Escherichia coli isolates of human and avian origin, Germany. Diagn Microbiol Infect Dis.

[39] Silva DAV, Dieckmann R, Makarewicz O, Hartung A, Bethe A, Grobbel M (2023). Biocide susceptibility and antimicrobial resistance of Escherichia coli isolated from swine feces, pork meat and humans in Germany. Antibiotics.

[40] Tu Z, Pang L, Lai S, Zhu Y, Wu Y, Zhou Q (2024). The hidden threat: comprehensive assessment of antibiotic and disinfectant resistance in commercial pig slaughterhouses. Sci Total Environ.

[41] Beshiru A, Igbinosa EO, Al Dahouk S, Dieckmann R, Neuhaus S (2025). Genetic diversity, biocide susceptibility and biofilm forming ability of Escherichia coli isolates from slaughterhouse environments in Nigeria. LWT.

[42] Mon‐On N, Surachetpong W, Mongkolsuk S, Sirikanchana K (2018). Roles of water quality and disinfectant application on inactivation of fish pathogenic Streptococcus agalactiae with povidone iodine, quaternary ammonium compounds and glutaraldehyde. J Fish Dis.

[43] Alvarado-Flores C, Encina-Montoya F, Tucca F, Vega-Aguayo R, Nimptsch J, Oberti C (2021). Assessing the ecological risk of active principles used currently by freshwater fish farms. Sci Total Environ.

[44] Gufe C, Karembera B, Marumure J, Makuvara Z (2022). Lead, silver nitrate and antibiotic resistance in bacteria isolated from Nile tilapia (Oreochromis niloticus) in anthropogenically polluted Lake Chivero, Zimbabwe. Cogent Food Agric.

[45] Ajewole OA, Ikhimiukor OO, Adelowo OO (2021). Heavy metals (Cu and Zn) contamination of pond sediment and co-occurrence of metal and antibiotic resistance in Escherichia coli from Nigerian aquaculture. Int J Environ Stud.

[46] Sarkar MM, Rohani MF, Hossain MAR, Shahjahan M (2022). Evaluation of heavy metal contamination in some selected commercial fish feeds used in Bangladesh. Biol Trace Elem Res.

[47] Bowler P, Murphy C, Wolcott R (2020). Biofilm exacerbates antibiotic resistance: is this a current oversight in antimicrobial stewardship? Antimicrob Resist Infect Control.

[48] Qian W, Li X, Yang M, Liu C, Kong Y, Li Y (2022). Relationship between antibiotic resistance, biofilm formation, and biofilm-specific resistance in Escherichia coli isolates from Ningbo, China. Infect Drug Resist.

[49] Obe T, Nannapaneni R, Schilling W, Zhang L, Kiess A (2021). Antimicrobial tolerance, biofilm formation, and molecular characterization of Salmonella isolates from poultry processing equipment. J Appl Poult Res.

[50] Ture M, Kilic MB, Altinok I (2021). Relationship between heavy metal accumulation in fish muscle and heavy metal resistance genes in bacteria isolated from fish. Biol Trace Elem Res.

[51] Nguyen TH, Nguyen HD, Le MH, Nguyen TT, Nguyen TD, Nguyen DL (2023). Efflux pump inhibitors in controlling antibiotic resistance: outlook under a heavy metal contamination context. Molecules.

[52] Tong C, Hu H, Chen G, Li Z, Li A, Zhang J (2021). Disinfectant resistance in bacteria: mechanisms, spread, and resolution strategies. Environ Res.

[53] Jiang X, Yu T, Liu L, Li Y, Zhang K, Wang H (2017). Examination of quaternary ammonium compound resistance in Proteus mirabilis isolated from cooked meat products in China. Front Microbiol.

